# Relationship between Inflammatory Cytokines and Indices of Cardiac Dysfunction following Intense Endurance Exercise

**DOI:** 10.1371/journal.pone.0130031

**Published:** 2015-06-12

**Authors:** Andre La Gerche, Warrick J. Inder, Timothy J. Roberts, Maria J. Brosnan, Hein Heidbuchel, David L. Prior

**Affiliations:** 1 Baker IDI Heart and Diabetes Institute, Melbourne, Australia; 2 St Vincent’s Hospital Melbourne, Fitzroy, Australia; 3 University Hospitals Leuven, KU Leuven, Belgium; 4 St Vincent’s Department of Medicine, University of Melbourne, Fitzroy, Australia; 5 University of Queensland, and Princess Alexandra Hospital, Brisbane, Australia; 6 Hasselt University and Heart Center, Jessa Hospital, Hasselt, Belgium; University of the Balearic Islands, SPAIN

## Abstract

**Objectives:**

Pro-inflammatory cytokines have been noted to increase following exercise but their relationship to exercise-induced cardiac dysfunction has not previously been investigated. We sought to evaluate whether exercise-induced cardiac dysfunction was associated with increases in cytokines, particularly the pro-inflammatory cytokines IL-1β, IL-12p70 and TNFα, which have been most implicated in cardiac pathology.

**Methods:**

40 well-trained endurance athletes underwent evaluation prior to and immediately following one of four endurance sporting events ranging from 3 to 11 hours duration. Cytokines (IL-1β, IL-6, IL-8, IL-10, IL-12p70 and TNFα) were analyzed by flow cytometry from serum samples collected within 50 minutes of race completion. Cardiac troponin (cTnI) and B-type natriuretic peptide were combined with an echocardiographic assessment of cardiac function, and a composite of cTnI > 0.04 μg/L, BNP increase > 10 ng/L and a decrease in right ventricular ejection (RVEF) > 10% were prospectively defined as evidence of myocardial dysfunction.

**Results:**

Relative to baseline, IL-6 IL-8 and IL-10 increased 8.5-, 2.9-, and 7.1-fold, respectively, P<0.0001. Thirty-one (78%), 19 (48%) and 18 (45%) of the athletes met the pre-specified criteria for significant cTnI, BNP and RVEF changes, respectively. TNFα, IL-12p70 were univariate predictors of ΔRVEF and ΔBNP whilst none of the anti-inflammatory cytokines were significantly associated with these measures. Ten athletes (25%, all athletes competing in the endurance event of longest duration) met criteria for exercise-induced myocardial dysfunction. In these 10 athletes with myocardial dysfunction, as compared to those without, there was significantly greater post-race expression of the pro-inflammatory cytokines IL-12p70 (8.1±3.8pg/ml vs. 2.5±2.6pg/ml, P<0.0001) and TNFα (6.5±3.1pg/ml vs. 2.0±2.5pg/ml, P<0.0001).

**Conclusion:**

Cardiac dysfunction following intense endurance exercise was associated with increased expression of pro-inflammatory cytokines. This does not prove a causal relationship but provides rationale for further investigations into whether inflammation mediates exercise-induced myocardial dysfunction.

## Introduction

Increases in circulating leukocytes and cytokines have been well characterized during bouts of intense exercise [1–4] but their role in cardiovascular health and disease remains unclear. Moderate exercise is associated with improved health outcomes and it has been argued that anti-inflammatory cytokines such as Interleukins 6, 8 and 10 (IL-6, IL-8 and IL-10) may potentiate some of these benefits [5]. The benefits of more extreme bouts of intense prolonged exercise are less established [6] and the inflammatory response may also differ from that of mild/ moderate exercise [1, 3, 7] In animal models, a single bout of extreme exercise was shown to elicit myocardial damage associated with acute inflammatory myocardial infiltrates [8]. More recently, Benito et al. observed an increase in inflammatory and pro-fibrotic markers in the atria and right ventricle of rats following 16 weeks of intense physical training which was associated with a greater propensity to ventricular arrhythmias [9]. Analogous changes have been observed in humans. Single bouts of extreme exercise result in acute myocardial dysfunction and release of cardiac dysfunction biomarkers [10–13] whilst chronic exercise has been associated with pro-arrhythmic RV remodeling [14, 15]. However, direct myocardial evaluation is seldom justifiable in these athletes and so the contribution of inflammation to exercise-induced cardiac dysfunction is unknown.

The association between exercise and inflammatory cytokines is complex. It has been well demonstrated that exercise evokes an inflammatory response but the particular cytokines involved are expressed in a different profile to that of other systemic inflammatory states such as sepsis [16]. In sepsis, there is a marked and rapid increase in tumor necrosis factor alpha (TNFα) followed by the interleukins IL-6, IL-1ra and IL-10. In contrast, exercise promotes an early and profound increase in IL-6 but not TNFα. Pedersen et al. have coined the term “myokine” following the localization of IL-6 expression to skeletal muscle and have argued that its release during exercise is critical in suppressing cytokines that are more directly involved in tissue injury and inhibition of cellular metabolism (TNFα, IL-1 and IL-12) [16]. To summarize a complex and evolving understanding of the role of the cytokines in exercise and disease, it may be reasonable to group cytokines into anti-inflammatory (IL-6, IL-8 and IL-10 amongst others) and pro-inflammatory (TNFα, IL-12 and IL-1β amongst others).

Associations between inflammatory cytokines and myocardial disease have been most extensively studied in patients with congestive heart failure. IL-6 and TNFα have been demonstrated to increase with the severity of heart failure and predict mortality [17]. It has been demonstrated that both an excess or deficiency of IL-6 and/or TNFα may induce myotoxicity, fibrosis and cardiac dilation [18–20] leading to the hypothesis that inflammation orchestrates repair and regeneration following a metabolic stress, but that an over-zealous inflammatory response will promote damage [21]. Such a theory could apply to endurance exercise whereby extreme bouts could promote either transient dysfunction and/or regeneration. The fact that endurance exercise is associated with myocyte hypertrophy and hyperplasia would suggest that the dominant exercise stimulus is regenerative. However, the degree to which more extreme exercise and greater inflammatory responses contribute to adaptive and maladaptive cardiac regeneration have not been evaluated in humans.

We hypothesized that following intense endurance exercise, cardiac dysfunction may be associated with a predominantly pro-inflammatory cytokine response in well-trained athletes.

## Methods

### Subjects

The endurance athlete cohort enrolled for this study has been described previously [22]. In brief, volunteers were sought through advertisements to local triathlon clubs. The first 40 endurance athletes who met the following three criteria were invited to participate in the study: 1) they were well-trained (defined as >10 hours of intense training per week) and well-performed (having finished within the first 25% of the field in a recent endurance event), 2) they had no cardiac symptoms or cardiac risk factors and 3) they were planning to compete in 1 of 4 nominated events (a marathon, endurance triathlon, alpine cycling race and an ultra-triathlon). The distances, number of competitors and completion times for each endurance event are detailed in Table 1. Written informed consent was obtained from all subjects and the protocol was approved by the St Vincent’s Hospital Human Research Ethics Committee in accordance with the Declaration of Helsinki.

**Table 1:** Summary of endurance race details.

Race	Sports	Distance (km)	No. of participants (average finishing time)	No. of study subjects/ average finishing time	Ambient temperature (°C)
Marathon	Running	42.2	2616 (3 hrs 58 mins ±37 mins)	7 (2 hrs 59 mins ±30 mins) *	16–20
Long-triathlon	Swim/ ride/ run	1.9/ 90/ 21.1	988 (5 hrs 22 mins ± 37 mins)	11 (5 hrs 24 mins ± 25 mins)	18–31
Alpine cycling	Cycling	207	2400 (10 hrs 24 mins ± 50 mins)	9 (8 hrs 5 mins ± 42mins) *	24–36
Ultra-triathlon	Swim/ ride/ run	3.8/ 180/ 42.2	1411 (12 hrs 8 mins ± 1hr 37 mins)	13 (10 hrs 52 mins ±1 hr 16 mins)*	17–28

*p<0.01 for comparison between subjects and overall competitor finishing times

### Procedures

Athletes were studied at 2 time points: 1) at *baseline* in the three weeks prior to the endurance event during regular training but following 2–3 days of relatively light training, and 2) immediately (10–50 minutes) following the endurance sporting event–*post-race*. The investigations performed were: blood tests for cardiac and inflammatory biomarkers, echocardiography and electrocardiography.

#### Cardiac and inflammatory biomarkers

Full blood was used for quantification of B-type natriuretic peptide (BNP) using a point-of-care immunoassay (Triage, Biosite Incorporated, San Diego, CA, USA) with a lower detection limit of 5 ng/L and for full blood count. The remaining samples were immediately centrifuged and plasma and serum samples stored at -80°C until analysis of sodium (Na), creatinine (Cr), osmolality, cytokines, cTnI and CK.

Cardiac troponin I (cTnI) was measured using an AxSYM cTnI assay (Abbott Laboratories, Abbott Park, IL, USA) for which the lower limit of detection was 0.015 and the lower 99th percentile of a normal reference population (URL) of 0.04 μg/L.

Concentrations of IL-8, IL-1β, IL-6, IL-10, TNFα, and IL-12p70 were quantified from serum samples using a multiplex cytometric bead array kit (BD-Biosciences, San Diego, CA). The kit utilizes beads with specific fluorescent intensities which have been coated with antibodies for the 6 different cytokines. The beads were then incubated with the subjects’ sera for 30 minutes and then amplified fluorescence detection by flow cytometry was analyzed using commercial software (BD-Biosciences, San Diego, CA) to obtain concentration values. The limit of blanc, defined as the corresponding concentration at two standard deviations above the median fluorescence of 20 replicates of the negative control (0 pg/ml), was IL-8 = 3.6 pg/ml, IL-1β = 7.2 pg/ml, IL-6 = 2.5 pg/ml, IL-10 = 3.3 pg/ml, TNFα = 3.7 pg/ml and IL-12p70 = 1.9 pg/ml.

#### Echocardiography

Baseline and post-race echocardiography was performed with the subject lying supine on their left side using a Vivid 7 Dimension echocardiograph (GE Vingmed Ultrasound, Horten, Norway). At least six full-volume 3-D data-sets (at least 3 of the RV and LV respectively) were acquired over five cardiac cycles during breath-hold. LV and RV volumes were then measured off-line using customized software (TomTec software, Germany) as previously described [23]. The average from three volume analyses from three separate acquisitions was used. At baseline, we validated these measures against cardiac magnetic resonance imaging and demonstrated good agreement between the two techniques (data not included in this report). Ejection fraction was quantified as (end-diastolic volume–end-systolic volume)/ end-diastolic volume. Detailed results of comprehensive echocardiographic results have been presented previously [22].

#### Definition of cardiac dysfunction

Cardiac dysfunction was prospectively defined as being present if all three of the following conditions were met: 1) post-race cTnI > 0.04 μg/L as a marker of myocardial damage by international guidelines [24], 2) post-race BNP increase > 10 ng/L as a marker of pathology in previous studies [25, 26] and 3) a relative decrease in RVEF > 10.6% of baseline representing a change of greater than 1.5 x standard deviation at baseline. We prospectively chose a decrease in RV ejection fraction (rather than LV ejection fraction) because we, and others, have demonstrated that prolonged endurance exercise preferentially affects RV function [10, 13, 27, 28] and, conversely, LV ejection fraction is minimally affected, if at all [29].

### Statistical analyses

Normal Gaussian distribution of continuous variables was tested using a Kolmogorov-Smirnov test. Baseline and post-race comparisons were performed using a paired samples t-test or a Wilcoxon signed-rank test as appropriate depending on whether the data was normally distributed. A chi-square test was used for comparison of categorical values. To assess the association between exercise-induced changes in biomarkers of myocardial dysfunction and cytokines, a stepwise linear regression model was used after assessment of significant colinearity. Statistical analysis was performed using IBM SPSS statistics 20 software. A two-tailed P-value of <0.05 was considered significant.

## Results

Subject characteristics according to endurance race grouping are presented in Table 2. Athletes in the four events had similar baseline measures of cardiac function and body type. The only differences were that athletes competing in the long triathlon were younger, and alpine cyclists older, than the mean. VO_2_max was also unequally represented across groups but these differences were not apparent when corrected for age and gender differences (predicted VO_2_). Ultra triathlon competitors performed more weekly training.

**Table 2:** Athlete demographics.

	Overall	Marathon	Long- triathlon	Alpine cycling	Ultra- triathlon	p-value
*n*	40	7	11	9	13	
Age (yrs)	37 ± 8	38 ± 3	33 ± 7	44 ± 9	34 ± 8	0.014
Male (%)	90	86	91	78	100	0.378
BMI (kg/m^2^)	23.6 ± 1.9	22.3 ± 1.6	24.0 ± 2.1	23.9 ± 2.1	23.5 ± 1.3	0.306
BSA (m^2^)	1.9 ± 0.2	1.9 ± 0.1	1.9 ± 0.2	2.0 ± 0.2	1.9 ± 0.1	0.918
VO_2_ max (ml/kg/min)	57.4 ± 6.4	55.5 ± 3.3	58.0 ± 9.2	53.2 ± 2.5	60.4 ± 5.0	0.046
Predicted VO_2_ (%)	146 ± 18	142 ± 8	141 ± 20	154 ± 20	148 ± 18	0.36
Training (years)	10 ± 9	13 ± 8	6 ± 5	12 ± 14	11 ± 9	0.277
Training (hrs/wk)	16.3±5.1	14 ± 6	14 ± 3	13 ± 4	21 ± 5	<0.0001

BMI, body mass index; BSA, body surface area (Dubois formula)

Underlined values signify those which differ from the mean

### Prevalence of myocardial dysfunction

Cardiac troponin was detectable in nine athletes (23%) at baseline, noting that testing was performed amidst a busy training schedule, and in all athletes post-race (p<0.0001 for comparison, Table 3).

**Table 3:** Cytokines, blood counts and biochemistry after short and endurance exercise as compared with baseline.

	Baseline	Post-race	p-value
*Measures of Cardiac Dysfunction*			
cTnI (μg/L)	0.010 ± 0.03	0.14 ± 0.17	<0.0001
cTnI > 0.04 μg/L (n, %)	0	31, 78%	<0.0001
BNP (ng/L)	13.2 ±14.2	25.4 ± 21	0.002
ΔBNP > 10 ng/L (n, %)	-	19, 48%	
RVEF (%)	51.0 ± 3.6	46.4 ± 6.5	<0.0001
ΔRVEF > -10.6% (n, %)	-	18, 45%	
‘Myocardial dysfunction’ (n, %)	0	10, 25%	0.0007
*Inflammatory cytokines*			
IL-6 (pg/ml)	3.91 ± 3.81	33.35 ± 24.01	<0.0001
IL-8 (pg/ml)	6.90 ± 5.41	19.81 ± 10.62	<0.0001
IL-10 (pg/ml)	2.85 ± 3.20	20.18 ± 22.52	<0.0001
IL-1β (pg/ml)	3.62 ± 3.39	3.95 ± 3.82	0.444
IL-12p70 (pg/ml)	3.81 ± 4.00	3.81 ± 3.69	0.829
TNFα (pg/ml)	2.73 ± 3.80	3.39 ± 3.85	0.161
*Hematology*			
Hb (g/l)	138.6 ± 7.6	149.1 ± 8.4	<0.0001
Haematocrit (%)	40.6 ± 2.4	44.1 ± 2.5	<0.0001
WCC (x10^9^/l)	5.81 ± 1.43	15.17 ± 3.84	<0.0001
Platelets (x10^9^/l)	250.5 ± 43.9	308.4 ± 55.5	<0.0001
*Biochemistry*			
Na (mmol/l)	138.4 ± 1.9	140.9 ± 2.9	0.002
K (mmol/l)	4.22 ± 0.24	4.93 ± 0.88	0.001
Creatinine (μmol/l)	79.9 ± 7.6	111.3 ± 19.2	<0.0001
CK (U/L)	305 ± 208	1001± 1006	<0.0001
Osmolality (mmol/kg)	285.8 ± 5.0	291.6 ± 7.4	0.002

In assessing the pre-specified definition of myocardial dysfunction, post-race cTnI was greater than 0.04 μg/L in 31 athletes (marathon 86%, long triathlon 100%, alpine cycling 33% and ultra-triathlon 85%, p = 0.003 for difference in proportions). There was also disproportionate representation in BNP increases across groups with 19 athletes (48%) fulfilling the definition of an increase >10 ng/L. The highest frequency was seen in the ultra-triathlon event (marathon 14%, long triathlon 27%, alpine cycling 22%, ultra-triathlon 92%, p<0.0001). RVEF decreased by >10.6% (1.5 x SD) in 18 subjects (marathon 29%, long triathlon 27%, alpine cycling 33%, ultra-triathlon 77%, p = 0.046). Ten athletes (25%) met all three criteria, all of which were ultra-triathletes (77% of this group).

### Relation between inflammatory cytokines and myocardial dysfunction

Inflammatory, hematological and biochemical measures at baseline and post-race are detailed in Table 3. IL-6, IL-8 and IL-10 increased 8.5-, 2.9-, and 7.1-fold respectively following the endurance race, whilst there was no significant change in IL-12p70, TNFα or IL-1β. Consistent with an acute inflammatory response, there was a large increase in white cell count (WCC) and a modest increase in platelets in the post-race setting. Hematocrit and serum osmolality increased compared to baseline, consistent with a degree of hemoconcentration. Creatine kinase (CK) levels also increased significantly, suggesting a degree of exercise-induced skeletal muscle injury.

**Fig 1:**
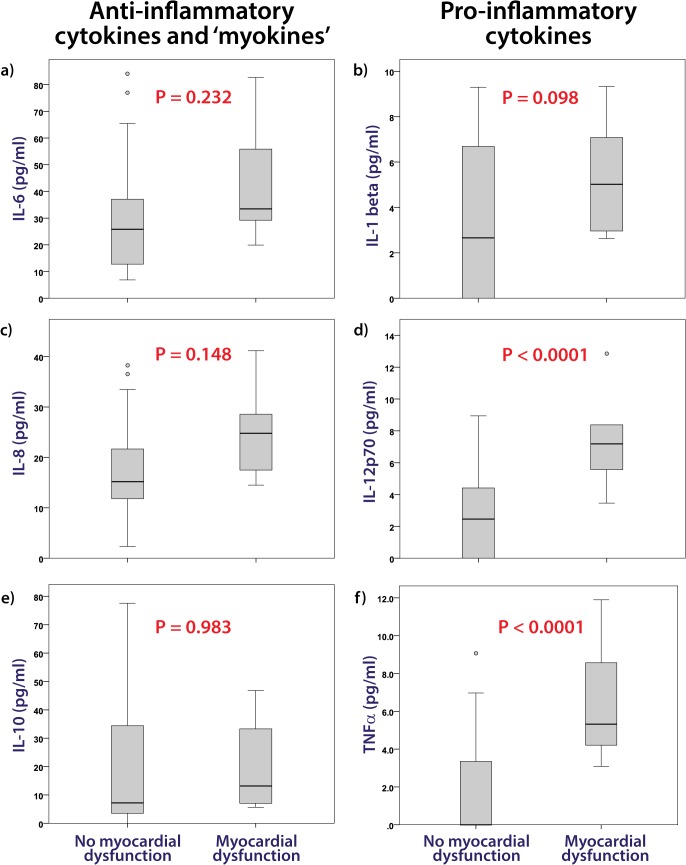
Post endurance race cytokine concentrations comparing athletes according to the presence or absence of myocardial dysfunction. The post-endurance exercise levels of the pro-inflammatory cytokines IL-6 (a) and IL-8 (c) and the anti-inflammatory cytokine IL-10 (e) did not differ according to the presence or absence of myocardial dysfunction whereas the pro-inflammatory cytokines IL-12p70 (d) and TNFα (f) were greater in those with cardiac dysfunction and a similar trend was evident for IL-1β (b).

Ten of the 40 endurance athletes were identified as having post-race myocardial dysfunction according to the prospective definition combining functional and biochemical measures. Thus it was possible to compare the inflammatory response in those with and without demonstrable cardiac dysfunction. Following the endurance race there was no difference in hematological values between those 10 athletes with myocardial dysfunction and those without (Hb, 147 ± 5 vs. 150 ± 9, p = 0.279; WCC, 16.2 ± 3.6 vs. 14.8, p = 0.315; platelets, 310 ± 34 vs. 308 ± 61, p = 0.876). IL-6 and IL-8 increased after endurance exercise (Table 3) but there was no difference in levels according to the presence or absence of myocardial dysfunction (Fig 1A and 1C). Similarly the anti-inflammatory cytokine IL-10 increased to a similar extent in both groups (Fig 1E). However, a number of potentially important cytokines were expressed to a greater extent in those subjects with myocardial dysfunction. IL-12p70 and TNFα were greater in athletes with post-race cardiac dysfunction (Fig 1D and 1F) whilst a similar trend in IL-1β was not significant (Fig 1B).

**Fig 2:**
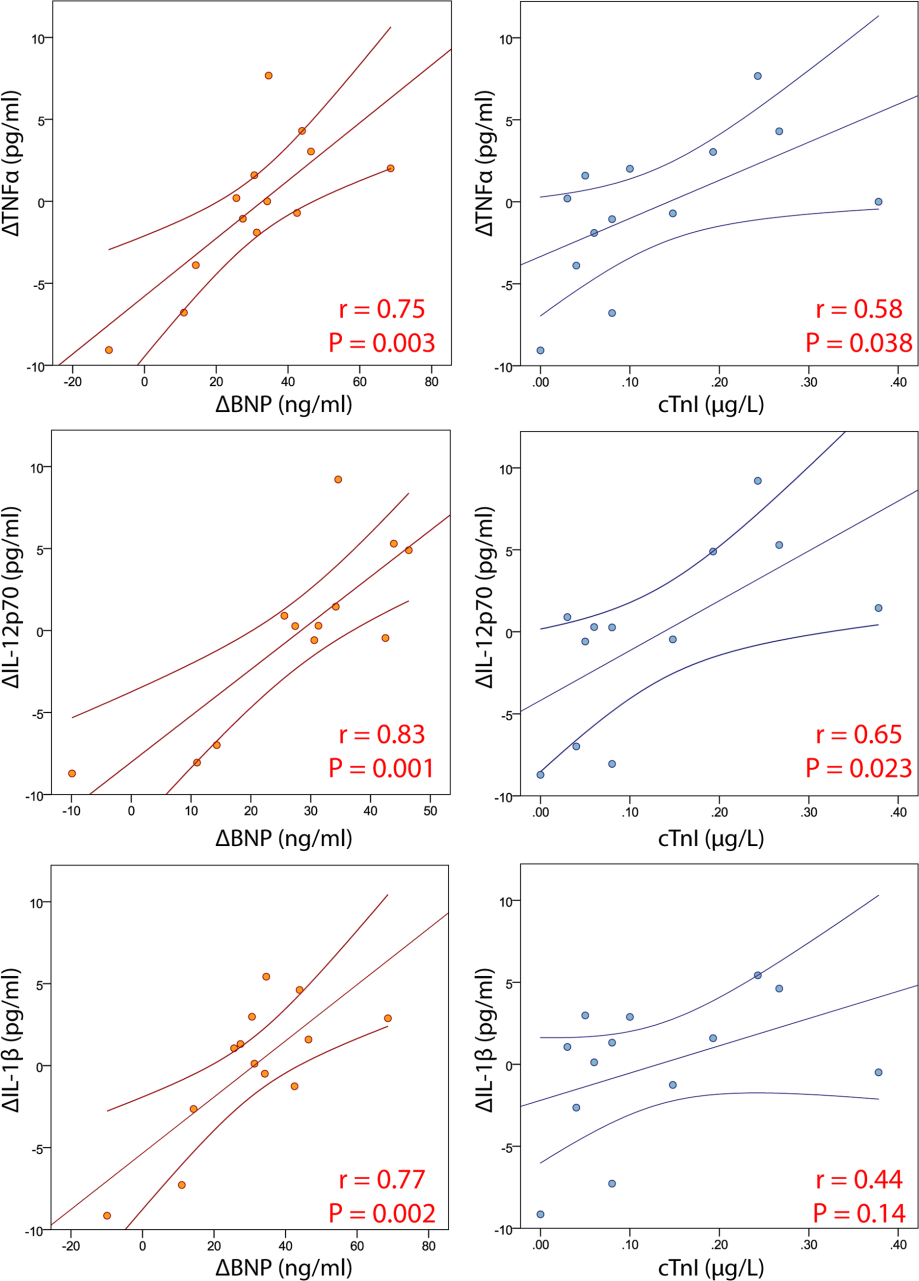
Correlations between pro-inflammatory cytokines and biochemical markers of myocardial dysfunction. Dot plot graphs and Pearson correlations are presented for the relationships between BNP and cTnI (as markers of myocardial dysfunction) and the pro-inflammatory cytokines in the 13 athletes competing in the ultra-endurance triathlon.

Strong correlations were observed between changes in BNP and changes in TNFα, IL-12p70 and IL-1β in the 13 athletes competing in the ultra-endurance triathlon. Modest correlations were similarly observed between changes in cTnI and both TNFα and IL-12p70 (Fig 2).

**Fig 3:**
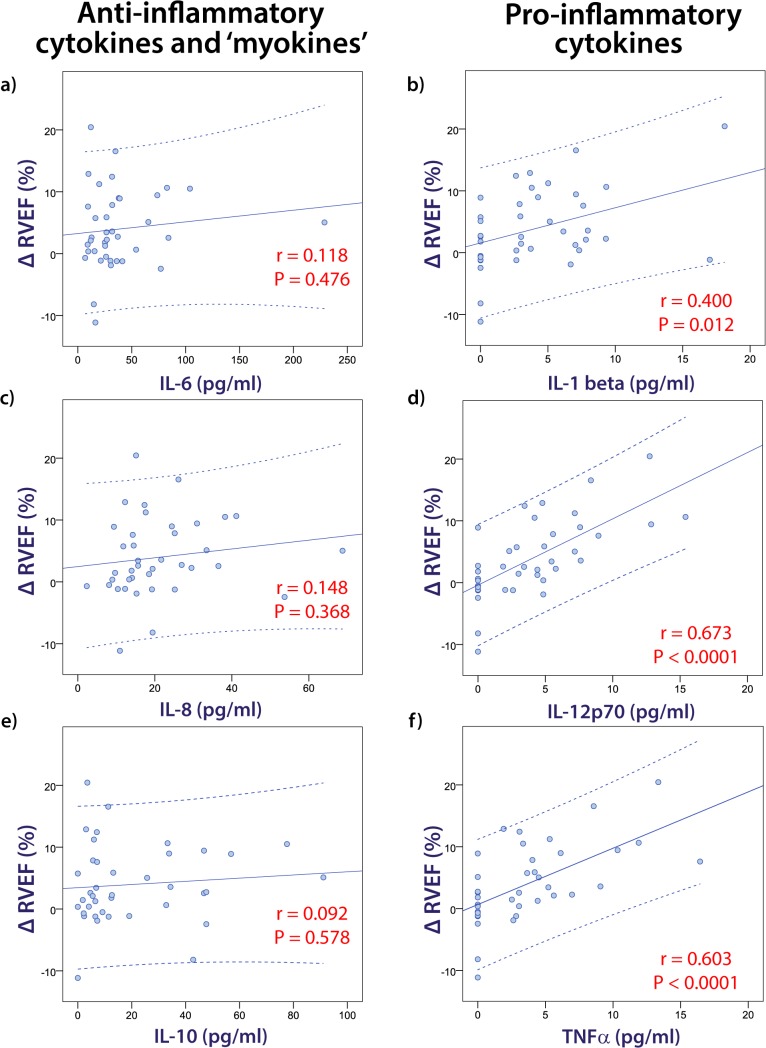
Correlations between pro-inflammatory cytokines and change in right ventricular ejection fraction (ΔRVEF). Dot plot graphs and Pearson correlations are presented for the relationships between ΔRVEF and cytokines.

In addition to the categorical analysis of athletes according to the pre-specified definition of myocardial injury, the relationship between cytokines and cardiac dysfunction was assessed using multiple regression analyses. IL-12p70, TNFα and IL-1β were all univariate predictors of the change in RVEF from baseline to the post-race examination (r = 0.673, r = 0.603 and r = 0.400 respectively, P < 0.05) whereas there was no significant association between ΔRVEF and any of the anti-inflammatory cytokines (Fig 3). With the important caveat that there was significant colinearity between the three pro-inflammatory cytokines, IL-12p70 was the only independent predictor of ΔRVEF on multivariate regression analysis, explaining 45% of the variance (P < 0.0001). Similarly, TNFα (r = 0.447, P = 0.004) and IL-12p70 (r = 0.419, P = 0.008) were independent predictors of ΔBNP, with TNFα remaining as the only independent predictor after multivariate analysis. None of the cytokines were significantly associated with ΔcTnI.

## Discussion

Exercise-induced pro-inflammatory responses have been well documented but this is the first study to describe an association between cardiac dysfunction resulting from intense-endurance exercise and increases in cytokines implicated in cardiovascular disease. Myocardial inflammation and necrosis has been demonstrated in murine models of extreme exercise [8, 9, 30] but, until now, there has not been data to support this premise in humans. Whilst this study may add some weight to this premise, a causative link between systemic inflammation and myocardial dysfunction cannot be proven in this current study.

### Inflammation and cytokine responses to exercise

The exercise-induced increases in IL-6, IL-8 and IL-10 are consistent with previous investigations [31]. Increases in IL-6 have been demonstrated with short bouts of exercise [32] and after ultra-endurance exercise [33], although most studies have evaluated durations of exercise in between these extremes. The extent, temporal profile and the ratio between the pro-inflammatory IL-6 and IL-8 and the anti-inflammatory IL-10 in our current study are all in keeping with the concept of a balanced immunological response to exercise [5]. Outside the context of endurance exercise, some studies have reported an association between increased levels of IL-6 and cardiac disease. Melendez et al. provided the most direct evidence for potential pathogenesis by demonstrating myocardial hypertrophy and fibrosis in rats following IL-6 infusion [34]. Intriguingly, Marcus et al. described an association between atrial fibrillation, increased IL-6 expression and IL-6 1744CC genotype in a cross-sectional study of patients with known coronary artery disease [35]. This is of particular interest given the increased prevalence of atrial fibrillation that has been described amongst endurance athletes [36, 37] and raises the possibility that inflammatory mediators may be important arrhythmic modulators. How then does one assimilate studies which link IL-6, cardiac fibrosis and arrhythmias with the seemingly opposing literature which links IL-6 (either alone or in combination with increases in IL-8 and IL-10) to a host of cardiovascular benefits including enhanced insulin sensitivity, lipolysis and reduced basal inflammatory states [5, 16, 31]? It is very possible that this apparent contradiction may be explained by the temporal profile of the cytokine expression. Much the same as physiological markers such as heart rate, it may be that exercise-related increases lead to down-regulation of basal cytokine levels. Thus, chronic and sustained increases in IL-6, IL-8 and IL-10 may be deleterious to cardiovascular health and best prevented by exercise. This is supported by the observation that cytokine levels are indeed lowest in well-trained athletes and increased in sedentary subjects [38]. Thus, our finding that increases in IL-6, IL-8 and IL-10 was not associated with cardiac dysfunction is consistent with the concept of healthy exercise-induced expression of these cytokines.

Whereas it may be argued that exercise-induced IL-6 expression may have a number of beneficial effects in attenuating chronic low-grade inflammation [5], the release of IL-12p70, IL-1β and TNFα have been more definitively associated with cardiac and vascular pathology. TNFα plays an early prominent role in infectious inflammatory responses and those resulting from burns but most studies have found that exercise does not stimulate TNFα, IL-12p70 or IL-1β release [5]. There is, however, some evidence that TNFα expression may increase following exercise of more extreme intensity and duration. Ostrowski et al. observed a small but significant increase in TNFα following a marathon run (mean exercise time 3 hours, 27 minutes) [2] but, in contrast, Limongelli et al. found no increase in TNFα when 20 elite athletes were evaluated following a short bout (approximately 10 minutes) of maximal intensity cycling. We observed variable increases in IL-12p70, IL-1β and TNFα according to whether athletes showed evidence of myocardial dysfunction. In the 10 athletes meeting the pre-specified definition of myocardial dysfunction there was significant exercise-induced expression of TNFα and IL-12p70 whilst this was not the case in those who did not manifest cardiac dysfunction. Indirectly, this may reflect the duration of exercise because it was notable that cardiac dysfunction was found in 10 of the 13 ultra-endurance athletes (77%) but none of the athletes completing shorter events. Furthermore, when assessing those 13 ultra-endurance athletes we observed a strong association between biochemical markers of myocardial dysfunction (BNP and cTnI) and the pro-inflammatory cytokines (Fig 2). Thus, our findings raise the important hypothesis that more prolonged intense exercise may be associated with a pro-inflammatory response, although it is not possible to assess whether inflammation is a cause, effect or is independent of tissue damage. Histological examinations of skeletal muscle biopsies have clearly demonstrated inflammation and necrosis following prolonged exercise [33] and our current data provide indirect evidence to suspect similar changes in cardiac muscle. Whilst damage to skeletal muscle may have little impact on muscular integrity and function, even microscopic damage to the myocardium has the theoretical risk of creating a pro-arrhythmic substrate. A causal link between extreme exercise, inflammation and arrhythmias (both atrial and ventricular) has been demonstrated in rats [36, 39] and our current data suggests that this is also a plausible line of inquiry in humans.

TNFα, IL-12p70 and IL-1β differ from the ‘myokines’ discussed previously in that there is no evidence to suggest a protective or beneficial role. Rather, these cytokines correlate with mortality in heart failure [17] and TNFα over-expression causes severe heart failure in animal models [19]. It is also intriguing to note that TNFα-induced cardiac dysfunction is predominantly mediated through pressure overload states [40, 41] and this effect may be greater for the RV than LV [42]. Therefore, our observed association between pro-inflammatory cytokines and exercise-induced RV dysfunction may be of significance. Such changes could be consistent with the hypothesis of greater RV wall stress during exercise inducing RV dysfunction via an inflammatory process [6].

There are a number of important limitations to be considered in the interpretation of this study. Firstly, blood samples for cytokine analysis were taken within 50 minutes of race completion. It is possible that the observed increases in pro-inflammatory cytokines observed in the ultra-endurance athletes was not because of the observed myocardial dysfunction but because of the temporal profile of exercise-induced cytokine expression. It is possible that TNFα may also have been elevated in those athletes completing shorter races but that the peak occurred many hours after race completion. The only way of assessing this possibility would have been to perform cytokine assays on multiple samples taken over a prolonged period during recovery. The logistics and expense of such an undertaking were beyond the scope of this project. Secondly, serum concentrations of these cytokines do not enable localization of their source. Given that post-endurance exercise skeletal muscle injury far exceeds that of cardiac dysfunction, it is more likely that the increase in serum cytokine expression predominantly reflects skeletal muscle injury. As stated previously, profound skeletal muscle inflammation has previously been observed after endurance exercise [33] and our finding of a significant increase in CK supports a degree of skeletal muscle injury. However, this would not explain the increases in troponin, BNP and the RV dysfunction which are all cardiac specific. Finally, we chose a prospective definition for myocardial dysfunction which combined biochemical and functional abnormalities. This definition is somewhat arbitrary but given that there is no pre-existing definition of myocardial dysfunction which is relevant to the post-race setting, we felt that the chosen criteria were logical and robust. Furthermore, the results of this categorical definition of myocardial dysfunction concur with the findings of significant associations with pro-inflammatory cytokines using continuous data in the multiple regressions.

## Conclusion

Following intense endurance exercise we found an association between greater expression of pro-inflammatory cytokines and myocardial dysfunction. This does not prove a causal relationship but provides rationale for further investigations into whether inflammation mediates exercise-induced myocardial dysfunction.

## References

[1] Neubauer O, Konig D, Wagner KH. Recovery after an Ironman triathlon: sustained inflammatory responses and muscular stress. Eur J Appl Physiol. 2008;104(3):417–26. Epub 2008/06/13. 10.1007/s00421-008-0787-6 . 18548269

[2] Ostrowski K, Rohde T, Asp S, Schjerling P, Pedersen BK. Pro- and anti-inflammatory cytokine balance in strenuous exercise in humans. The Journal of physiology. 1999;515 (Pt 1):287–91. Epub 1999/02/02. . 9925898 10.1111/j.1469-7793.1999.287ad.x PMC2269132

[3] Peake JM, Suzuki K, Hordern M, Wilson G, Nosaka K, Coombes JS. Plasma cytokine changes in relation to exercise intensity and muscle damage. Eur J Appl Physiol. 2005;95(5–6):514–21. Epub 2005/09/10. 10.1007/s00421-005-0035-2 . 16151834

[4] Wardyn GG, Rennard SI, Brusnahan SK, McGuire TR, Carlson ML, Smith LM, et al Effects of exercise on hematological parameters, circulating side population cells, and cytokines. Exp Hematol. 2008;36(2):216–23. Epub 2008/01/22. doi: S0301-472X(07)00617-0 [pii] 10.1016/j.exphem.2007.10.003 . 18206729

[5] Pedersen BK. The anti-inflammatory effect of exercise: its role in diabetes and cardiovascular disease control. Essays Biochem. 2006;42:105–17. Epub 2006/12/06. doi: bse0420105 [pii] 10.1042/bse0420105 . 17144883

[6] La Gerche A, Heidbuchel H. Can intensive exercise harm the heart? You can get too much of a good thing. Circulation. 2014;130(12):992–1002. Epub 2014/09/17. 10.1161/circulationaha.114.008141 . 25223770

[7] Serrano E, Venegas C, Escames G, Sanchez-Munoz C, Zabala M, Puertas A, et al Antioxidant defence and inflammatory response in professional road cyclists during a 4-day competition. J Sports Sci. 2010;28(10):1047–56. Epub 2010/08/06. doi: 925189793 [pii] 10.1080/02640414.2010.484067 . 20686993

[8] Chen Y, Serfass RC, Mackey-Bojack SM, Kelly KL, Titus JL, Apple FS. Cardiac troponin T alterations in myocardium and serum of rats after stressful, prolonged intense exercise. J Appl Physiol. 2000;88(5):1749–55. . 10797139 10.1152/jappl.2000.88.5.1749

[9] Benito B, Gay-Jordi G, Serrano-Mollar A, Guasch E, Shi Y, Tardif JC, et al Cardiac arrhythmogenic remodeling in a rat model of long-term intensive exercise training. Circulation. 2011;123(1):13–22. Epub 2010/12/22. doi: CIRCULATIONAHA.110.938282 [pii] 10.1161/CIRCULATIONAHA.110.938282 . 21173356

[10] La Gerche A, Connelly KA, Mooney DJ, MacIsaac AI, Prior DL. Biochemical and functional abnormalities of left and right ventricular function after ultra-endurance exercise. Heart (British Cardiac Society). 2008;94(7):860–6. Epub 2007/05/08. doi: hrt.2006.101063 [pii] 10.1136/hrt.2006.101063 . 17483127

[11] Neilan TG, Januzzi JL, Lee-Lewandrowski E, Ton-Nu TT, Yoerger DM, Jassal DS, et al Myocardial injury and ventricular dysfunction related to training levels among nonelite participants in the Boston marathon. Circulation. 2006;114(22):2325–33. Epub 2006/11/15. doi: CIRCULATIONAHA.106.647461 [pii] 10.1161/CIRCULATIONAHA.106.647461 . 17101848

[12] Rifai N, Douglas PS, O'Toole M, Rimm E, Ginsburg GS. Cardiac troponin T and I, echocardiographic [correction of electrocardiographic] wall motion analyses, and ejection fractions in athletes participating in the Hawaii Ironman Triathlon. The American journal of cardiology. 1999;83(7):1085–9. . 10190525 10.1016/s0002-9149(99)00020-x

[13] Trivax JE, Franklin BA, Goldstein JA, Chinnaiyan KM, Gallagher MJ, deJong AT, et al Acute cardiac effects of marathon running. J Appl Physiol. 2010;108(5):1148–53. Epub 2010/02/13. doi: 01151.2009 [pii] 10.1152/japplphysiol.01151.2009 . 20150567

[14] Heidbuchel H, Hoogsteen J, Fagard R, Vanhees L, Ector H, Willems R, et al High prevalence of right ventricular involvement in endurance athletes with ventricular arrhythmias. Role of an electrophysiologic study in risk stratification. European heart journal. 2003;24(16):1473–80. . 12919770 10.1016/s0195-668x(03)00282-3

[15] La Gerche A, Robberecht C, Kuiperi C, Nuyens D, Willems R, de Ravel T, et al Lower than expected desmosomal gene mutation prevalence in endurance athletes with complex ventricular arrhythmias of right ventricular origin. Heart (British Cardiac Society). 2010;96(16):1268–74. Epub 2010/06/08. doi: hrt.2009.189621 [pii] 10.1136/hrt.2009.189621 . 20525856

[16] Pedersen BK, Febbraio MA. Muscle as an endocrine organ: focus on muscle-derived interleukin-6. Physiol Rev. 2008;88(4):1379–406. Epub 2008/10/17. doi: 88/4/1379 [pii] 10.1152/physrev.90100.2007 . 18923185

[17] Deswal A, Petersen NJ, Feldman AM, Young JB, White BG, Mann DL. Cytokines and cytokine receptors in advanced heart failure: an analysis of the cytokine database from the Vesnarinone trial (VEST). Circulation. 2001;103(16):2055–9. . 11319194 10.1161/01.cir.103.16.2055

[18] Kinugawa K, Takahashi T, Kohmoto O, Yao A, Aoyagi T, Momomura S, et al Nitric oxide-mediated effects of interleukin-6 on [Ca2+]i and cell contraction in cultured chick ventricular myocytes. Circulation research. 1994;75(2):285–95. . 7518362 10.1161/01.res.75.2.285

[19] Bryant D, Becker L, Richardson J, Shelton J, Franco F, Peshock R, et al Cardiac failure in transgenic mice with myocardial expression of tumor necrosis factor-alpha. Circulation. 1998;97(14):1375–81. . 9577949 10.1161/01.cir.97.14.1375

[20] Banerjee I, Fuseler JW, Intwala AR, Baudino TA. IL-6 loss causes ventricular dysfunction, fibrosis, reduced capillary density, and dramatically alters the cell populations of the developing and adult heart. Am J Physiol Heart Circ Physiol. 2009;296(5):H1694–704. Epub 2009/02/24. doi: 00908.2008 [pii] 10.1152/ajpheart.00908.2008 19234091 PMC2685341

[21] Jiang B, Liao R. The paradoxical role of inflammation in cardiac repair and regeneration. J Cardiovasc Transl Res. 2010;3(4):410–6. Epub 2010/06/19. 10.1007/s12265-010-9193-7 . 20559773

[22] La Gerche A, Burns AT, Mooney DJ, Inder WJ, Taylor AJ, Bogaert J, et al Exercise-induced right ventricular dysfunction and structural remodelling in endurance athletes. European heart journal. 2012;33(8):998–1006. Epub 2011/12/14. doi: ehr397 [pii] 10.1093/eurheartj/ehr397 . 22160404

[23] Niemann PS, Pinho L, Balbach T, Galuschky C, Blankenhagen M, Silberbach M, et al Anatomically oriented right ventricular volume measurements with dynamic three-dimensional echocardiography validated by 3-Tesla magnetic resonance imaging. Journal of the American College of Cardiology. 2007;50(17):1668–76. Epub 2007/10/24. doi: S0735-1097(07)02462-X [pii] 10.1016/j.jacc.2007.07.031 . 17950149

[24] Thygesen K, Alpert JS, White HD. Universal definition of myocardial infarction. Journal of the American College of Cardiology. 2007;50(22):2173–95. Epub 2007/11/27. doi: S0735-1097(07)02957-9 [pii] 10.1016/j.jacc.2007.09.011 . 18036459

[25] Foote RS, Pearlman JD, Siegel AH, Yeo KT. Detection of exercise-induced ischemia by changes in B-type natriuretic peptides. Journal of the American College of Cardiology. 2004;44(10):1980–7. . 15542280 10.1016/j.jacc.2004.08.045

[26] Sabatine MS, Morrow DA, de Lemos JA, Omland T, Desai MY, Tanasijevic M, et al Acute changes in circulating natriuretic peptide levels in relation to myocardial ischemia. Journal of the American College of Cardiology. 2004;44(10):1988–95. . 15542281 10.1016/j.jacc.2004.07.057

[27] Mousavi N, Czarnecki A, Kumar K, Fallah-Rad N, Lytwyn M, Han SY, et al Relation of biomarkers and cardiac magnetic resonance imaging after marathon running. The American journal of cardiology. 2009;103(10):1467–72. Epub 2009/05/12. doi: S0002-9149(09)00288-4 [pii] 10.1016/j.amjcard.2009.01.294 . 19427448

[28] Douglas PS, O'Toole ML, Hiller WD, Reichek N. Different effects of prolonged exercise on the right and left ventricles. Journal of the American College of Cardiology. 1990;15(1):64–9. Epub 1990/01/01. . 2295743 10.1016/0735-1097(90)90176-p

[29] Middleton N, Shave R, George K, Whyte G, Hart E, Atkinson G. Left ventricular function immediately following prolonged exercise: A meta-analysis. Med Sci Sports Exerc. 2006;38(4):681–7. Epub 2006/05/09. doi: 10.1249/01.mss.0000210203.10200.12 00005768-200604000-00011 [pii]. . 16679983 10.1249/01.mss.0000210203.10200.12

[30] Guasch E, Benito B, Qi X, Cifelli C, Naud P, Shi Y, et al Atrial fibrillation promotion by endurance exercise: demonstration and mechanistic exploration in an animal model. Journal of the American College of Cardiology. 2013;62(1):68–77. Epub 2013/04/16. 10.1016/j.jacc.2013.01.091 . 23583240

[31] Febbraio MA, Pedersen BK. Muscle-derived interleukin-6: mechanisms for activation and possible biological roles. Faseb J. 2002;16(11):1335–47. . 12205025 10.1096/fj.01-0876rev

[32] Nielsen HB, Secher NH, Christensen NJ, Pedersen BK. Lymphocytes and NK cell activity during repeated bouts of maximal exercise. Am J Physiol. 1996;271(1 Pt 2):R222–7. Epub 1996/07/01. . 8760224 10.1152/ajpregu.1996.271.1.R222

[33] Marklund P, Mattsson CM, Wahlin-Larsson B, Ponsot E, Lindvall B, Lindvall L, et al Extensive inflammatory cell infiltration in human skeletal muscle in response to an ultraendurance exercise bout in experienced athletes. J Appl Physiol (1985). 2013;114(1):66–72. Epub 2012/10/30. 10.1152/japplphysiol.01538.2011 . 23104690

[34] Melendez GC, McLarty JL, Levick SP, Du Y, Janicki JS, Brower GL. Interleukin 6 mediates myocardial fibrosis, concentric hypertrophy, and diastolic dysfunction in rats. Hypertension. 2010;56(2):225–31. Epub 2010/07/08. 10.1161/HYPERTENSIONAHA.109.148635 20606113 PMC2921860

[35] Marcus GM, Whooley MA, Glidden DV, Pawlikowska L, Zaroff JG, Olgin JE. Interleukin-6 and atrial fibrillation in patients with coronary artery disease: data from the Heart and Soul Study. Am Heart J. 2008;155(2):303–9. Epub 2008/01/25. 10.1016/j.ahj.2007.09.006 18215601 PMC2247366

[36] Abdulla J, Nielsen JR. Is the risk of atrial fibrillation higher in athletes than in the general population? A systematic review and meta-analysis. Europace. 2009;11(9):1156–9. Epub 2009/07/28. 10.1093/europace/eup197 . 19633305

[37] La Gerche A, Schmied CM. Atrial fibrillation in athletes and the interplay between exercise and health. European heart journal. 2013. 10.1093/eurheartj/eht265 . 23884920

[38] Fischer CP, Berntsen A, Perstrup LB, Eskildsen P, Pedersen BK. Plasma levels of interleukin-6 and C-reactive protein are associated with physical inactivity independent of obesity. Scand J Med Sci Sports. 2007;17(5):580–7. Epub 2006/11/02. 10.1111/j.1600-0838.2006.00602.x . 17076827

[39] Mont L. Arrhythmias and sport practice. Heart (British Cardiac Society). 2010;96(5):398–405. Epub 2010/03/04. doi: 96/5/398 [pii] 10.1136/hrt.2008.160903 . 20197369

[40] Kapadia SR, Oral H, Lee J, Nakano M, Taffet GE, Mann DL. Hemodynamic regulation of tumor necrosis factor-alpha gene and protein expression in adult feline myocardium. Circulation research. 1997;81(2):187–95. Epub 1997/08/01. . 9242179 10.1161/01.res.81.2.187

[41] Sun M, Chen M, Dawood F, Zurawska U, Li JY, Parker T, et al Tumor necrosis factor-alpha mediates cardiac remodeling and ventricular dysfunction after pressure overload state. Circulation. 2007;115(11):1398–407. Epub 2007/03/14. doi: CIRCULATIONAHA.106.643585 [pii] 10.1161/CIRCULATIONAHA.106.643585 . 17353445

[42] Markel TA, Crisostomo PR, Wang M, Herrmann JL, Abarbanell AM, Meldrum DR. Right ventricular TNF resistance during endotoxemia: the differential effects on ventricular function. Am J Physiol Regul Integr Comp Physiol. 2007;293(5):R1893–7. Epub 2007/08/24. doi: 00359.2007 [pii] 10.1152/ajpregu.00359.2007 . 17715182

